# Correction to: Endoscopic surgical treatment for rhinogenic contact point headache: systematic review and meta-analysis

**DOI:** 10.1007/s00405-021-06844-z

**Published:** 2021-05-07

**Authors:** Antonino Maniaci, Federico Merlino, Salvatore Cocuzza, Giannicola Iannella, Claudio Vicini, Giovanni Cammaroto, Jérome R. Lechien, Christian Calvo-Henriquez, Ignazio La Mantia

**Affiliations:** 1 Department of Medical and Surgical Sciences and Advanced Technologies “GF Ingrassia”, ENT Section, ENT Department of University of Catania, Via Santa Sofia, 95100 Catania, Italy; 2 Department of ‘Organi Di Senso’, University “Sapienza”, Rome, Italy; 3 Department of Head-Neck Surgery, Otolaryngology, Head-Neck, and Oral Surgery Unit, Morgagni Pierantoni Hospital, Forlì, Italy; 4 Research Committee of the Young Otolaryngologists, International Federations of ORL Societies, Paris, France; 5 Department of Human Anatomy and Experimental Oncology, School of Medicine, UMONS Research Institute for Health Sciences and Technology, University of Mons, Mons, Belgium; 6 Department of Otorhinolaryngology-Head and Neck Surgery, CHU Saint-Pierre, School of Medicine, Université Libre de Bruxelles, Brussels, Belgium; 7 Department of Otolaryngology-Head and Neck Surgery, Foch Hospital, (University of Paris-Saclay), Paris, France; 8 Task Force COVID-19 of the Young-Otolaryngologists of the International Federations of Oto-Rhino-Laryngological Societies (YO-IFOS), Department of Otolaryngology, Hospital Complex of Santiago de Compostela, Santiago de Compostela, Spain

## Correction to: European Archives of Oto-Rhino-Laryngology https://doi.org/10.1007/s00405-021-06724-6

In the original publication of the article, one of the author name was published incorrectly as “Jérome Lechien”. The correct name is “Jérome R. Lechien”.

The original article was updated.

